# *Wolbachia* distribution in selected beetle taxa characterized by PCR screens and MLST data

**DOI:** 10.1002/ece3.1641

**Published:** 2015-09-16

**Authors:** Rebekka Sontowski, Detlef Bernhard, Christoph Bleidorn, Martin Schlegel, Michael Gerth

**Affiliations:** ^1^ Molecular Evolution and Systematics of AnimalsInstitute for BiologyUniversity of LeipzigTalstrasse 33D‐04103LeipzigGermany; ^2^ German Centre for Integrative Biodiversity Research (iDiv) Halle‐Jena‐LeipzigDeutscher Platz 5d04103LeipzigGermany

**Keywords:** Buprestidae, Coleoptera, Dytiscidae, endosymbionts, Hydraenidae, Hydrophilidae

## Abstract

*Wolbachia* (Alphaproteobacteria) is an inherited endosymbiont of arthropods and filarial nematodes and was reported to be widespread across insect taxa. While *Wolbachia*'s effects on host biology are not understood from most of these hosts, known *Wolbachia*‐induced phenotypes cover a spectrum from obligate beneficial mutualism to reproductive manipulations and pathogenicity. Interestingly, data on *Wolbachia* within the most species‐rich order of arthropods, the Coleoptera (beetles), are scarce. Therefore, we screened 128 species from seven beetle families (Buprestidae, Hydraenidae, Dytiscidae, Hydrophilidae, Gyrinidae, Haliplidae, and Noteridae) for the presence of *Wolbachia*. Our data show that, contrary to previous estimations, *Wolbachia* frequencies in beetles (31% overall) are comparable to the ones in other insects. In addition, we used *Wolbachia* MLST data and host phylogeny to explore the evolutionary history of *Wolbachia* strains from Hydraenidae, an aquatic lineage of beetles. Our data suggest that *Wolbachia* from Hydraenidae might be largely host genus specific and that *Wolbachia* strain phylogeny is not independent to that of its hosts. As this contrasts with most terrestrial *Wolbachia*–arthropod systems, one potential conclusion is that aquatic lifestyle of hosts may result in *Wolbachia* distribution patterns distinct from those of terrestrial hosts. Our data thus provide both insights into *Wolbachia* distribution among beetles in general and a first glimpse of *Wolbachia* distribution patterns among aquatic host lineages.

## Introduction

*Wolbachia* is a genus of obligatory intracellular, inherited bacteria that is found in many arthropods and in filarial nematodes (Werren et al. 2008). Its impact on host biology is diverse and complex: while most distinguished for inducing reproductive modifications in their hosts (Stouthamer et al. 1999), *Wolbachia* exhibits a large array of phenotypes, ranging from mutualism (Hosokawa et al. 2010; Chrostek et al. 2013) to pathogenicity (Le Clec'h et al. 2012). In terrestrial arthropods, *Wolbachia* was estimated to be present in 40% of all species (Zug and Hammerstein 2012), thus making it the most successful endosymbiont on earth. One key to this success is the ability of *Wolbachia* strains to invade and adapt to new hosts, aside from being transmitted vertically from female to progeny. Although such horizontal transmissions are evident from a lack of cocladogenesis between *Wolbachia* and its hosts (O'Neill et al. 1992; Baldo et al. 2008), the underlying mechanisms are not fully understood. Parasitoids have been identified as potential drivers of horizontal *Wolbachia* transfer, and it has been hypothesized that successful *Wolbachia* establishment in novel hosts is correlated with both host ecology and host phylogeny (Russell et al. 2009; Stahlhut et al. 2010). However, determining the routes of *Wolbachia* invasions in natural arthropod populations is challenging, as patterns are usually blurred by regular gains and losses of *Wolbachia*, in addition to frequent horizontal transmissions (Baldo et al. 2008; Gerth et al. 2013).

Molecular classification of main *Wolbachia* lineages follows a “supergroup” scheme (Zhou et al. 1998), with the great majority of arthropod *Wolbachia* belonging to supergroups A, B, and, more rarely, supergroup F (Duron et al. 2008). On a finer scale, a multilocus sequence typing (MLST) system allows discrimination of *Wolbachia* strains within supergroups (Baldo et al. 2006). For strain definition, five conserved housekeeping genes are employed, similar to MLST schemes developed for other microorganisms (Maiden et al. 1998). Previous PCR screens covering a large spectrum of arthropod taxa or focussing on single taxa have revealed *Wolbachia* to be widespread in almost all hexapod orders (reviewed in Russell 2012). Surprisingly, little is known about *Wolbachia* in the most species‐rich order of insects, the beetles (Coleoptera). In addition to their taxonomic diversity, beetles are also ecologically diverse, having invaded all major habitats on earth and displaying a large variety of lifestyles (Dettner and Peters 2011). Endosymbionts in other arthropods profoundly impact host biology, allow the exploitation of new ecological niches, and contribute to their diversification (Moran 2007; Brucker and Bordenstein 2012). Conceivably, similar microbe/host interactions remain to be uncovered in Coleoptera, which comprise a large part of arthropod species.

As yet, specific endosymbiont surveys in beetles were mainly focussed on economically important weevils (Heddi et al. 1999; Conord et al. 2008; Lachowska et al. 2010; Toju and Fukatsu 2010; Russell 2012; Merville et al. 2013) and chrysomelids (Clark et al. 2001; Kondo et al. 2011), and on male‐killing bacteria in coccinellids (Weinert et al. 2007). However, these taxa represent just a small fraction of around 386,000 described beetle species from 176 families (Grimaldi and Engel 2005; Slipinski et al. 2011), and it is therefore unclear whether beetles in general are rarely infected by *Wolbachia*, as current data suggest (Russell 2012), or whether this observation reflects a sampling bias, as most beetle taxa were so far not screened for *Wolbachia*. In this study, we therefore determined the distribution of *Wolbachia* endosymbionts in 128 species from seven families of beetles so far not investigated for the presence of *Wolbachia* (Buprestidae, Hydraenidae, Dytiscidae, Hydrophilidae, Gyrinidae, Haliplidae, and Noteridae) by means of a PCR screen. Buprestidae are typically associated with wood, some being important pests, for example, the emerald ash borer. In contrast, all other beetles investigated in this study are aquatic and are of special interest because data on *Wolbachia* in aquatic hosts are generally scarce. In addition to the PCR screen, we used a MLST approach to gain insights into the evolutionary history of *Wolbachia* within Hydraenidae, an aquatic beetle lineage with around 1600 described species (Slipinski et al. 2011).

Hydraenidae (minute moss beetles) are considered to be “true water beetles”; that is, most of the adult stage is spent submerged in freshwater (Jäch 1998). Adults are tiny (1–3 mm) and can be found in a variety of aquatic habitats, including stagnant water, running water, and seepages, whereas the larvae are largely terrestrial (Jäch and Balke 2007). However, lifestyles of most hydraenid larval instars are unknown, as the larvae have been described for only 1% of the species. Furthermore, the distinction between terrestrial and aquatic lifestyles is often difficult, if not impossible, for minute beetle larvae living at the land–water margin (Jäch and Balke 2007).

Here, we were interested in whether *Wolbachia* distribution among Hydraenidae follows a random pattern as found in many terrestrial arthropods or whether the aquatic lifestyle has a distinct impact on this pattern. Although the precise factors influencing *Wolbachia* distribution are not known in most terrestrial arthropod host systems, it is conceivable that they are different to the ones governing transmission routes under water; for example, only very few parasitoids (as potential vectors of endosymbionts) are aquatic (Godfray 1994). To test this prediction, we employed *Wolbachia* MLST data and reconstructed a phylogeny of Hydraenidae hosts using nuclear and mitochondrial markers. Next, we tested whether host phylogeny is nonrandomly associated with *Wolbachia* strain phylogeny and *vice versa*. Our data provide insights into the evolution and distribution of *Wolbachia* in Coleoptera and may thus be regarded as a basis for future studies on beetle/*Wolbachia* interactions.

## Materials and Methods

### Insect collection, DNA extraction, and PCR conditions

Beetles were collected between 2001 and 2012 during various field trips from diverse places, mostly in Europe (Table S1). We used DNA extractions that were performed during previous molecular phylogenetic studies on these beetles (Korte et al. 2004; Bernhard et al. 2005, 2006, 2009; Karagyan et al. 2011) well as novel DNA extracts acquired from whole specimens with a protocol modified from Gustincich et al. (1991, Table S1). Altogether, 155 individuals of 128 beetle species from 7 families were surveyed for *Wolbachia*. To test for the presence of *Wolbachia*, we used PCR conditions and primers (*ftsz_F1, ftsz_R1*) from Baldo et al. (2006). To verify the presence of *Wolbachia*, all amplified fragments of *Wolbachia* cell division protein gene (*ftsZ*) were sequenced in both forward and reverse direction by GATC Biotech, Konstanz, Germany. Our approach may have resulted in underestimations of the actual prevalence of *Wolbachia*, because (1) only a small number of individuals per species could be included in the screen, and hence, rare infections were likely to be overlooked; and (2) we cannot exclude the possibility of false‐negative PCRs. However, these caveats hold true for most endosymbiont screens.

*Wolbachia* MLST profiling of strains extracted from 14 Hydraenidae species followed standard protocols and primers (Baldo et al. 2006; http://pubmlst.org/wolbachia/). In order to test for potential correlations between *Wolbachia* and host phylogeny, we also reconstructed the phylogeny of 27 Hydraenidae species (+2 outgroup species) investigated in this study based on the nuclear loci 18S ribosomal RNA gene (*18S*) and 28S ribosomal RNA gene (*28S*), and the mitochondrial gene cytochrome c oxidase subunit 1 (*COI*). The dataset was compiled from NCBI GenBank and complemented by novel sequences (29 species altogether, Table S2) amplified using the protocols and primers of Ribera et al. (2010), Medlin et al. (1988), and Simon et al. (1994) for *18S*,* 28S,* and *COI*, respectively. All sequences generated in this study were submitted to NCBI GenBank under accession numbers KT199105–KT199229 (Tables S1 and S2).

### Sequence editing and phylogenetic analyses

Single sequences were manually corrected in BioEdit 7.1.11.0 (Hall 1999) and assembled using the implemented greedy CAP algorithm (Huang 1992). All loci were aligned separately with Mafft 7.215, using the L‐INS‐i strategy (Katoh and Standley 2013). *Wolbachia* supergroup affiliation was determined by reconstructing a maximum likelihood tree of all *ftsz* sequences from this study and sequences from NCBI GenBank with RAxML 8.1.15 (Stamatakis 2014) under the GTR+G model. *Wolbachia* MLST loci were aligned and trimmed using templates from PubMLST database (http://pubmlst.org/wolbachia). We reconstructed *Wolbachia* phylogeny with ClonalFrame 1.2, a Bayesian software that infers clonal relationships from MLST data and incorporates recombination events (Didelot and Falush 2007). Three independent runs were performed with 500,000 generations each and a burn‐in of 20%. Convergence of runs was assessed with the methods of Gelman and Rubin (1992) implemented in ClonalFrame. All post‐burn‐in trees were used to build a majority‐rule consensus tree and to infer posterior probabilities from clade frequencies. In addition, we inferred a maximum likelihood tree of *Wolbachia* strains by concatenating the five MLST loci into a supermatrix with FasConCat 1.0 (Kück and Meusemann 2010) and performing a combined tree search and bootstrapping with 1000 pseudoreplicates in RAxML under the GTR+G model.

For nuclear and mitochondrial loci of Hydraenidae, we determined the best fitting nucleotide substitution models by calculating log likelihoods of 88 models with IQ‐TREE 1.2.1 (Nguyen et al. 2014) and ranking them by AIC (Akaike 1974). As GTR+G+I was favoured for all partitions, we combined the single genes into a supermatrix. RAxML was used for tree search and bootstrapping (1000 pseudoreplicates). In addition, we used MrBayes 3.2.2 (Ronquist and Huelsenbeck 2003) to reconstruct Hydraenidae phylogeny. Two runs with 4 chains each were run for 500,000 generations and a burn‐in of 25%. Convergence of runs was assumed when split frequencies reached <0.01 and sampling size of parameters was considered sufficiently large (ESS values >100). A majority‐rule consensus tree was constructed from the post‐burn‐in samples, and posterior probabilities obtained from clade frequencies.

To test for potential nonrandom phylogenetic associations of *Wolbachia* strains and Hydraenidae hosts, we employed BaTS 1.0 (Parker et al. 2008). Briefly, this software uses three test statistics to evaluate whether a trait is nonrandomly distributed in a given phylogeny. It was therefore used to assess whether *Wolbachia* strains are randomly distributed in Hydraenidae, as in most terrestrial arthropod systems, or whether there is a phylogenetic determinant shaping this distribution. BaTS enables accounting for phylogenetic uncertainty, as the posterior distribution of trees (e.g., from a Bayesian analysis) is used instead of a single fixed topology. For our dataset, we tested both whether *Wolbachia* strains are nonrandomly distributed onto the Hydraenidae phylogeny and whether hydraenid hosts are nonrandomly distributed onto *Wolbachia* phylogeny. For both tests, we used the posterior sample of trees acquired in Bayesian analyses described above (Hydraenidae: MrBayes, *Wolbachia*: ClonalFrame). We then coded the corresponding traits (Hydraenidae: genus *Hydraena* or *Ochthebius*; for *Wolbachia*: supergroup A or B, or no *Wolbachia*) and ran BaTS using 1000 replicates each. An additional analysis was performed for the posterior sample from a MrBayes analysis of a reduced supermatrix containing only *Wolbachia*‐infected Hydraenidae hosts (*N* = 17).

In addition to these trait‐based tests, we also directly tested for congruence between *Wolbachia* and Hydraenidae trees, using ParaFit (Legendre et al. 2002; Poland and McCullough 2006) and PACo (Balbuena et al. 2013). Both methods provide test statistics to assess whether phylogenetic positions of corresponding hosts and symbionts are independent of each other. This is achieved via randomization of host–symbiont associations. As opposed to ParaFit, PACo (Procrustean Approach to Cophylogeny) allows to explicitly test the dependence of one phylogeny (here: *Wolbachia*) upon the other (Hydraenidae). As both tests require distance matrices of hosts and symbionts, we calculated genetic distances of the concatenated MLST dataset for *Wolbachia* strains and the concatenated nuclear and mitochondrial loci for Hydraenidae using the “dist.dna” function of the R package ape and the TN93 model (Paradis et al. 2004). Furthermore, we created patristic distance matrices from the best scoring maximum likelihood trees of both of these datasets using the function “cophenetic.phylo” as implemented in ape. ParaFit and PACo were performed within the R statistical environment (R Development Core Team 2012), using both types of distance matrices and 100,000 permutations each.

## Results

We tested 155 individuals from 128 species comprising seven beetle families (Buprestidae, Hydraenidae, Dytiscidae, Hydrophilidae, Gyrinidae, Haliplidae, and Noteridae) for the presence of *Wolbachia*. We found it in 31% of the tested species and in all of the seven families (Table 1). *Wolbachia* frequencies were uniform (14–21%) across the families with ≥12 included species, except for Hydraenidae, in which *Wolbachia* was found in a proportion of 63% of the tested species (Table 1). By maximum likelihood analysis of the obtained *ftsz* sequences together with sequences from databases, three distinct *Wolbachia* supergroups could be determined in our sample of beetles (Figure S1). While supergroup A was most common, and found in all beetle families (26/40 infected species), we also detected supergroup B in 12 species of Buprestidae, Hydraenidae, and Dytiscidae. Furthermore, supergroup F *Wolbachia* was present in two species of Buprestidae. We did not find evidence for the occurrence of multiple *Wolbachia* strains in any of the analyzed specimens.

**Table 1:** Distribution of *Wolbachia* in beetle families screened in this study

Family	Number of species (individuals) investigated	Proportion/number of *Wolbachia*‐positive species	Detected supergroups
Buprestidae	61 (78)	21% / 14	A, B, F
Hydraenidae	27 (29)	63% / 17	A, B
Dytiscidae	21 (25)	14% / 3	A, B
Hydrophilidae	12 (15)	17% / 2	A
Gyrinidae	3 (3)	33% / 1	A
Haliplidae	2 (3)	50% / 1	A
Noteridae	2 (2)	100% / 2	A
Total	128 (155)	31% / 40	

**Figure 1:**
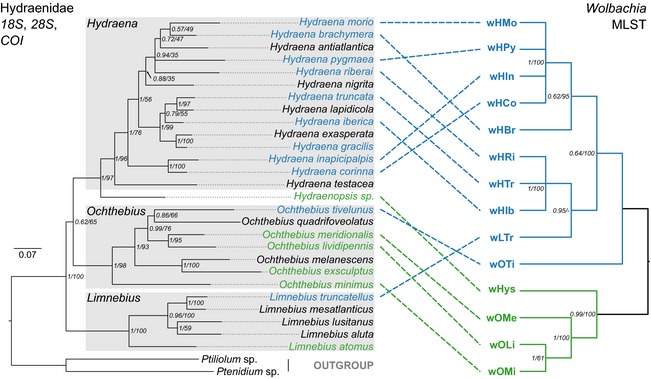
Phylogenetic patterns among Hydraenidae and corresponding *Wolbachia* strains. Left: Phylogenetic relationships among investigated Hydraenidae estimated with MrBayes based on *18S*,* 28S,* and *COI* sequences. Numbers on nodes correspond to posterior probabilities from Bayesian analysis/bootstrap values from RAxML analysis. Right: ClonalFrame phylogeny of *Wolbachia* strains from Hydraenidae. Numbers on nodes correspond to posterior probabilities from ClonalFrame analysis and bootstrap values from RAxML analysis. Host/*Wolbachia* associations are indicated by dashed lines. Blue color indicates supergroup A *Wolbachia* strains and Hydraenidae carrying supergroup A *Wolbachia, g*reen color marks supergroup B. Please note that MLST was not successful for three *Wolbachia* strains from Hydraenidae (*Hydraena gracilis*,*Ochthebius exsculptus*,*Limnebius atomus*), which are therefore not represented in the *Wolbachia* phylogeny.

Bayesian and maximum likelihood phylogenies of Hydraenidae based on *18S*,* 28S,* and *COI* were identical (Fig. 1). Each of the genera that included more than a single representative was recovered as monophyletic with high support (Fig. 1). However, within the genus *Hydraena*, relationships were only moderately supported by bootstrap from maximum likelihood analysis and posterior probabilities from Bayesian analysis, and some nodes could not be resolved with high confidence (Fig. 1). *Wolbachia* phylogenies based on five MLST loci were largely identical for both ClonalFrame and RAxML analyses, and most splits were highly supported (Fig. 1).

The topologies of Hydraenidae hosts and their corresponding *Wolbachia* strains were not completely congruent (Fig. 1). However, within *Hydraena*, all *Wolbachia* isolates were classified as supergroup A strains, and within *Ochthebius*, supergroup B was predominant (4/5 *Wolbachia* strains). BaTS analysis showed that the trait “host genus” is nonrandomly associated with *Wolbachia* phylogeny (Table S3). *Wolbachia* supergroups on the other hand were randomly associated with Hydraenidae phylogeny, but significantly associated with the reduced Hydraenidae tree comprising only *Wolbachia*‐infected species (Table S3). Furthermore, ParaFit and PACo analyses showed evidence for cophylogenetic patterns within our datasets: independence of *Wolbachia* and Hydraenidae phylogenies was statistically rejected for both genetic and patristic distance matrices by both approaches (*P*‐values 0.002–0.031, Table S4).

## Discussion

### Wolbachia prevalence and distribution in beetles

Our results show that *Wolbachia* is common in the investigated beetle families, with infection frequencies ranging from 14% to 63% for families with more than twelve sampled species, and 31% altogether (Table 1). Previous studies screening *Wolbachia* specifically in beetles are rare (Clark et al. 2001; Weinert et al. 2007; Lachowska et al. 2010), and a meta‐analysis covering these and other studies suggested that *Wolbachia* infections are generally rarer in beetles compared to other insect orders, while pointing out that this may be a sampling artifact (Russell 2012). Our data are in line with estimations of a general *Wolbachia* prevalence among arthropods (40–60%) and, due to our sampling design covering only one or a few individuals per species, may likely be an underestimation (Hilgenboecker et al. 2008; Zug and Hammerstein 2012). Notably, infection frequencies in Buprestidae, Dytiscidae, and Hydrophilidae seem to be lower than estimated by these meta‐analyses. In general, however, our survey suggests that *Wolbachia* is not more uncommon in Coleoptera than in other arthropods.

Furthermore, the distribution of *Wolbachia* supergroups in beetles is comparable to that described from other hosts. We found mostly supergroup A (64%), some supergroup B (31%), and only few supergroup F (5%) strains in our samples. Similar patterns are known from hymenopterans and dipterans, in which supergroup A is prevailing (Stahlhut et al. 2010; Gerth et al. 2011), while supergroup B is more common in lepidopterans (Russell et al. 2009). In most *Wolbachia* surveys to date, supergroup F was only very rarely encountered (Duron et al. 2008; Russell 2012), and it is the only lineage of *Wolbachia* that is found in both arthropods and nematodes (Ros et al. 2009). Adding to the peculiarities, supergroup F strains may be obligate mutualists (Hosokawa et al. 2010), and it is only distantly related to the other *Wolbachia* lineages infecting arthropods (Gerth et al. 2014). Our data do not allow speculating on potential impacts of *Wolbachia* onto their beetle hosts. However, as supergroup distribution patterns and general prevalence are similar to other arthropod groups, *Wolbachia*'s role in beetles is likely not very different to described ones, for example, reproductive parasitism (Werren et al. 2008) or protection from pathogens (Hedges et al. 2008).

### Wolbachia in Hydraenidae

*Wolbachia* from Hydraenidae were further closely investigated because (1) they showed the highest *Wolbachia* frequency of all analyzed beetle families; and (2) they are mainly aquatic and data on *Wolbachia* in aquatic arthropods are scarce. While *Wolbachia* has been detected in insects with aquatic larval stages, such as damselflies and dragonflies (Odonata), stone flies (Plecoptera), or various dipterans (Thipaksorn et al. 2003; Russell 2012), the only fully aquatic *Wolbachia* hosts reported so far, to our knowledge, are some crustacean species (Cordaux et al. 2001, 2012; Baltanás et al. 2007) and a single species of Dytiscidae (“diving beetles”) (Duron et al. 2008). We therefore aimed at investigating the evolutionary history of *Wolbachia* within Hydraenidae and at answering whether their aquatic lifestyle impacts *Wolbachia* strain distribution and results in markedly different patterns to the ones found in terrestrial systems. From several of these systems, *Wolbachia* strains were reported to be randomly distributed, with host phylogeny, ecology, and geography as factors that may influence this distribution (Russell et al. 2009; Stahlhut et al. 2010; Gerth et al. 2013).

In Hydraenidae, we found that the trait “host genus” is significantly associated with *Wolbachia* MLST phylogeny (Table S3). Furthermore, ParaFit and PACo analyses suggested that the phylogeny of analyzed *Wolbachia* strains is not independent from that of its hosts; that is, hydraenid phylogeny predicts the distribution of *Wolbachia* strains. This phylogenetic signal suggests that *Wolbachia* distribution among hydraenids is not random, and while horizontal transmissions and losses of *Wolbachia* have also likely occurred within the host genera (Fig. 1), this did not affect the signal potentially resulting from vertical *Wolbachia* transfer over evolutionary timescales. This is in contrast to what is known of *Wolbachia* strains from other arthropod hosts, in which these processes usually result in a blurred picture of *Wolbachia* transfers (Gerth et al. 2013). Two scenarios might explain this finding. (1) Horizontal movements of *Wolbachia* occur less often in aquatic environments than in terrestrial systems, for example, because there are fewer potential pathways of such transmissions under water. After *Wolbachia* supergroups A and B invaded Hydraenidae independently, they codiverged with their hosts. Because only few lateral transfers or losses occurred, these ancient invasion events are still reflected in the current distribution patterns (*Hydraena*: supergroup A, *Ochthebius*: supergroup B, Fig. 1) and in the correlation of host and *Wolbachia* phylogenies (Table S4). (2) *Wolbachia* dynamics are not different in aquatic environments. Horizontal movements or losses occur as frequently as in terrestrial systems, which would explain the seemingly random distribution of supergroup A strains within *Hydraena* (Fig. 1). The phylogenetic signal is maintained because supergroup A *Wolbachia* outperform other *Wolbachia* strains within the genus *Hydraena* and supergroup B *Wolbachia* are more successful in *Ochthebius* species. Given that *Wolbachia* supergroups A and B are ubiquitously spread, yet unevenly distributed, for example, between arthropod orders (Ros et al. 2009; Russell et al. 2009), competition between *Wolbachia* strains and differential adaptation to certain host environments can be expected. Both scenarios, however, remain speculative as long as the mechanisms of horizontal *Wolbachia* movements in terrestrial and aquatic systems are not understood and until additional aquatic *Wolbachia* hosts are investigated.

## Conclusions

It should be noted that our interpretations are based on a small dataset only: Hydraenidae comprise 1600 species (Slipinski et al. 2011), 900 of which were described from the genus *Hydraena* (Trizzino et al. 2013). Consequently, *Wolbachia* distribution patterns might look different when sampling a more representative sample of hydraenid species. Although our data suggest that *Wolbachia* infection dynamics in aquatic hosts might be distinct to the ones described from terrestrial hosts, data from further aquatic hosts are required to generalize our observations. Furthermore, we could show that *Wolbachia* prevalence and supergroup distribution in beetles (Coleoptera) are, in general, similar to patterns described from other insect orders.

## Conflict of Interest

None declared.

## Supporting information


**Figure S1.** Supergroup affiliation of investigated *Wolbachia* strains from beetles as determined via maximum likelihood analysis of *ftsz* sequences.

**Table S1.** List of all specimens screened for *Wolbachia* in this study. *Wolbachia* ftsz accession numbers are given for infected species.

**Table S2.** NCBI Accession numbers for all sequences employed for Hydraenidae and Wolbachia MLST phylogeny.

**Table S3.** Results of BaTS analyses for phylogeny‐trait associations.

**Table S4.** Results of ParaFit and PACo tests for congruence of Hydraenidae and *Wolbachia* phylogenies.
