# Supplementary figures and images for: Wolbachia distribution in selected beetle taxa characterized by PCR screens and MLST data
Source: Ecol Evol. 2015 Sep 16;5(19):4345–53. doi: 10.1002/ece3.1641 (PMC4667820; doi:10.1002/ece3.1641)

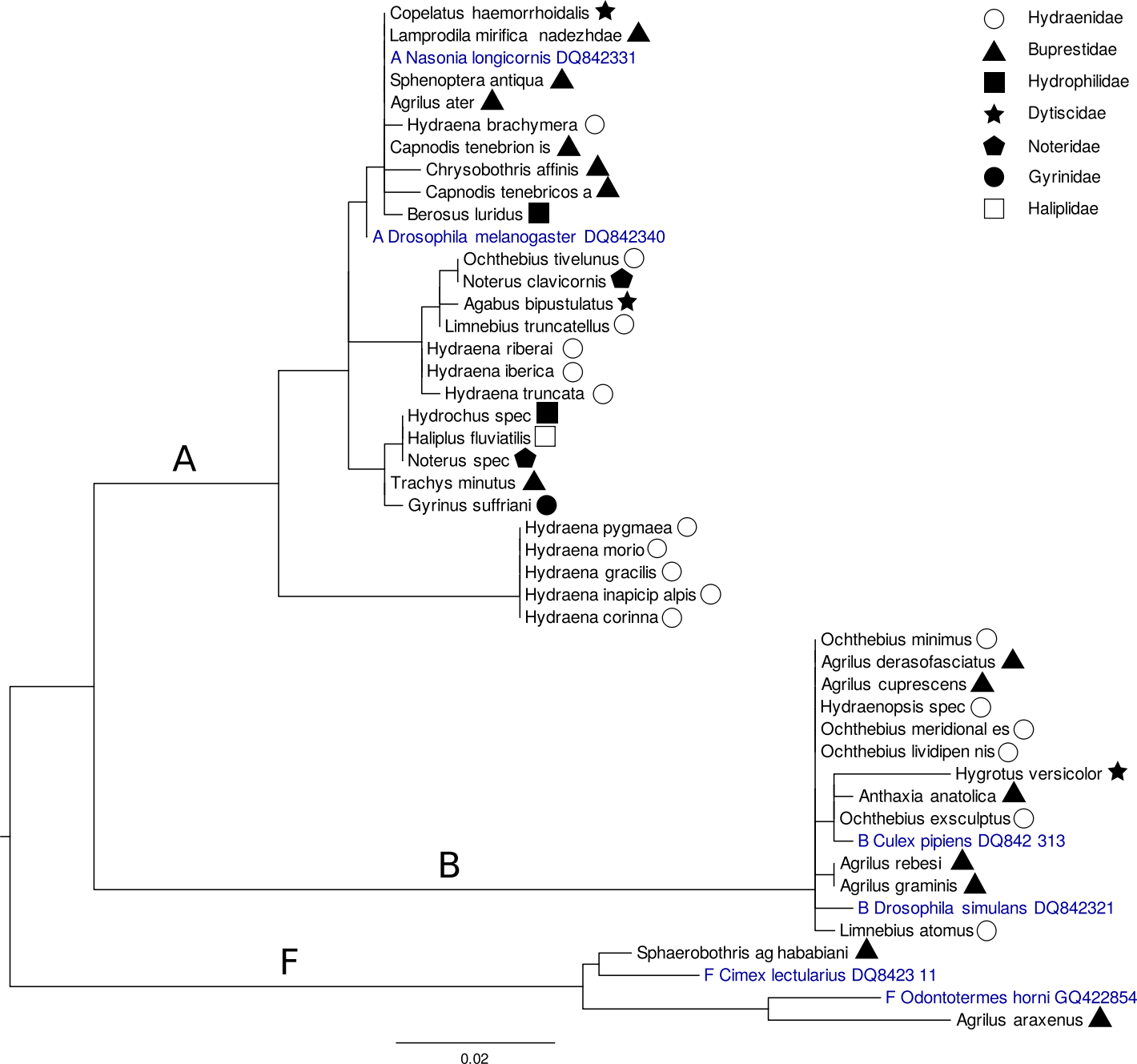

Supplement: Supplementary file 1 — Figure S1. Supergroup affiliation of investigated Wolbachia strains from beetles as determined via maximum likelihood analysis of ftsz sequences. [file ECE3-5-4345-s001.pdf]
