# Supplementary material for: Wolbachia distribution in selected beetle taxa characterized by PCR screens and MLST data
Source: Ecol Evol. 2015 Sep 16;5(19):4345–53. doi: 10.1002/ece3.1641 (PMC4667820; doi:10.1002/ece3.1641)
Supplement: Supplementary file 2 — Table S1. List of all specimens screened for Wolbachia in this study. Wolbachia ftsz accession numbers are given for infected species. [file ECE3-5-4345-s002.doc]

Table S1 List of all specimens screened for *Wolbachia* in this study. *Wolbachia* ftsz accession numbers are given for infected species.

|  |  |  |  |  |  |
| --- | --- | --- | --- | --- | --- |
|  |  |  |  |  |  |
| Species | Collecting place | Collecting time | *Wolbachia-infection* | NCBI accession number ftsZ | Reference / DNA source |
| **Buprestidae** |  |  |  |  | Karagyan et al. 2011 |
| *Acmaeodera (Ptychomus) polita* (Klug, 1829) | Yemen | n.n. | - |  | Karagyan et al. 2011 |
| *Acmaeodera degener* (Scopoli,1763) | Armenia | n.n. | - |  | Karagyan et al. 2011 |
| *Acmaeodera degener* (Scopoli,1763) | Southern France | n.n. | - |  | Karagyan et al. 2011 |
| *Acmaeodera flavolineata* Laporte & Gory, 1835 | Armenia | n.n. | - |  | Karagyan et al. 2011 |
| *Acmaeodera planidorsis* Semenov, 1895 | Armenia | n.n. | - |  | Karagyan et al. 2011 |
| *Acmaeoderella flavofasciata* (Piller & Mitterpacher, 1783) | Southern France | n.n. | - |  | Karagyan et al. 2011 |
| *Acmaeoderella mimonti* (Boieldieu, 1865) | Armenia | n.n. | - |  | Karagyan et al. 2011 |
| *Acmaeoderella plavilscikovi* (Obenberger, 1936) | Armenia | n.n. | - |  | Karagyan et al. 2011 |
| *Acmaeoderella vetusta* (Ménétriés, 1832) | Armenia | n.n. | - |  | Karagyan et al. 2011 |
| *Agrilus angustulus* (Illiger, 1803) | Austria (Leithagebirge) | 19/05/2002 | - |  | Bernhard et al. 2005 |
| *Agrilus angustulus* (Illiger, 1803) | France (Chamborigand) | 01/06/2002 | - |  | Bernhard et al. 2005 |
| *Agrilus araxenus* Iablokoff-Khnzorian, 1960 | Armenia | n.n. | F | KT199202 | Karagyan et al. 2011 |
| *Agrilus ater* (Linnaeus, 1767) | Southern France | n.n. | A | KT199186 | Bernhard et al. 2005 |
| *Agrilus biguttatus* (Fabricius, 1776) | Austria (Leithagebirge) | 19/05/2002 | - |  | Bernhard et al. 2005 |
| *Agrilus cuprescens* Ménétriés, 1832 | Austria (Leithagebirge) | 19/05/2002 | - |  | Bernhard et al. 2005 |
| *Agrilus cuprescens* Ménétriés, 1832 | Germany (Tübingen) | 12/06/2002 | B | KT199199 | Bernhard et al. 2005 |
| *Agrilus cyanescens* Ménétriés, 1832 | France (Chamborjand) | 01/06/2002 | - |  | Bernhard et al. 2005 |
| *Agrilus derasofasciatus* Lacerdaire, 1835 | Armenia | n.n. | B | KT199200 | Karagyan et al. 2011 |
| *Agrilus graminis* Gory & Laporte, 1837 | France (Chamborigand) | 01/06/2002 | B | KT199194 | Bernhard et al. 2005 |
| *Agrilus graminis* Gory & Laporte, 1837 | Germany (Neuenburg) | 20/06/1998 | - |  | Bernhard et al. 2005 |
| *Agrilus hyperici* (Creutzer, 1799) | Armenia | n.n. | - |  | Karagyan et al. 2011 |
| *Agrilus populneus* Schaefer, 1946 | Southern France | n.n. | - |  | Bernhard et al. 2005 |
| *Agrilus populneus* Schaefer, 1946 | Hungary (Bugac Pusta) | 23/05/2002 | - |  | Korte et al. 2004 |
| *Agrilus populneus* Schaefer, 1946 | Germany (Tübingen) | 12/06/2002 | - |  | Bernhard et al. 2005 |
| *Agrilus pratensis* Ratzeburg, 1839 | Germany | 12/06/2002 | - |  | Bernhard et al. 2005 |
| *Agrilus ribesi* Schaefer, 1946 | Germany (Tübingen) | 20/07/2002 | B | KT199193 | Bernhard et al. 2005 |
| *Agrilus sinuatus* (Olivier, 1790) | Germany (Tübingen) | 06/07/2002 | - |  | Bernhard et al. 2005 |
| *Agrilus sulcicollis* Lacordaire, 1835 | Germany (Tübingen) | 06/06/2002 | - |  | Bernhard et al. 2005 |
| *Agrilus viridis* (Linné, 1758) | n.n. | n.n. | - |  | Bernhard et al. 2005 |
| *Agrilus viridis* (Linné, 1758) | Germany (Tübingen) | 13/06/2002 | - |  | Bernhard et al. 2005 |
| *Agrilus viridis* (Linné, 1758) | Germany (Karlsruhe) | 24/04/2002 | - |  | Bernhard et al. 2005 |
| *Agrilus viridis* (Linné, 1758) | Germany (Heilbronn) | 23/04/2002 | - |  | Bernhard et al. 2005 |
| *Agrilus viridis* (Linné, 1758) | Germany (Heilbronn) | 23/04/2002 | - |  | Bernhard et al. 2005 |
| *Agrilus viridis* (Linné, 1758) | Germany (Karlsruhe) | 24/04/2002 | - |  | Bernhard et al. 2005 |
| *Agrilus viridis* (Linné, 1758) | Germany (Karlsruhe) | 24/04/2002 | - |  | Bernhard et al. 2005 |
| *Agrilus viridis* (Linné, 1758) | Germany (Karlsruhe) | 23/04/2002 | - |  | Bernhard et al. 2005 |
| *Agrilus viridis* (Linné, 1758) | n.n. | n.n. | - |  | Bernhard et al. 2005 |
| *Anthaxia amasina* Daniel, 1903 | Armenia | n.n. | - |  | Karagyan et al. 2011 |
| *Anthaxia anatolica* Chevrolat, 1838 | Armenia | n.n. | B | KT199201 | Karagyan et al. 2011 |
| *Anthaxia hungarica* (Scopoli, 1772) | Armenia | n.n. | - |  | Karagyan et al. 2011 |
| *Anthaxia mirabilis* Zhicharev, 1918 | Armenia | n.n. | - |  | Karagyan et al. 2011 |
| *Anthaxia nitidula* (Linné, 1758) | Austria (Leithagebirge) | 19/05/2002 | - |  | Bernhard et al. 2005 |
| *Anthaxia sp.* | Southern France | n.n. | - |  | Bernhard et al. 2005 |
| *Buprestis octoguttata (*Linnaeus, 1758) | Germany (Saarmund) | July 2012 | - |  | this study |
| *Buprestis octoguttata* (Linnaeus, 1758) | Germany (Saarmund) | July 2012 | - |  | this study |
| *Buprestis octoguttata* (Linnaeus, 1758) | Germany (Saarmund) | July 2012 | - |  | this study |
| *Capnodis miliaris* (Klug, 1829) | Armenia | n.n. | - |  | Karagyan et al. 2011 |
| *Capnodis tenebricosa* (Olivier, 1790) | Armenia | n.n. | A | KT199189 | Karagyan et al. 2011 |
| *Capnodis tenebrionis* (Linnaeus, 1761) | Southern France | n.n. | A | KT199185 | Karagyan et al. 2011 |
| *Chrysobothris affinis* (Fabricius, 1794) | Armenia | n.n. | A | KT199181 | Karagyan et al. 2011 |
| *Chrysobothris solieri* Gory & Laporte, 1893 | n.n. | n.n. | - |  | Karagyan et al. 2011 |
| *Coraebus rubi* (Linnaeus, 1767) | Armenia | n.n. | - |  | Karagyan et al. 2011 |
| *Habroloma nana* (Paykull, 1799) | Germany (Hirschhalde) | 11/06/2002 | - |  | Bernhard et al. 2005 |
| *Julodella globithorax* (Steven, 1830) | Armenia | n.n. | - |  | Karagyan et al. 2011 |
| *Julodis andreae* (Olivier, 1790) | Armenia | n.n. | - |  | Karagyan et al. 2011 |
| *Julodis faldermanni* Mannerheim, 1837 | Armenia | n.n. | - |  | Karagyan et al. 2011 |
| *Lamprodila (Scintillatrix) mirifica nadezhdae* (Semenov, 1909) | Armenia | n.n. | A | KT199188 | Karagyan et al. 2011 |
| *Meliboeus graminis* (Panzer, 1789) | Armenia | n.n. | - |  | Karagyan et al. 2011 |
| *Meliboeus robustus* Küster, 1852 | Armenia | n.n. | - |  | Karagyan et al. 2011 |
| *Perotis lugubris* (Fabricius, 1777) | Armenia | n.n. | - |  | Karagyan et al. 2011 |
| *Sphaerobothris aghababiani* Volkovitsh & Kalashian, 1998 | Armenia | n.n. | F | KT199203 | Karagyan et al. 2011 |
| *Sphenoptera antiqua* (Illger, 1803) | Armenia | n.n. | A | KT199187 | Karagyan et al. 2011 |
| *Sphenoptera artemisiae* Reitter, 1889 | Armenia | n.n. | - |  | Karagyan et al. 2011 |
| *Sphenoptera fallatrix* Obenberger, 1927 | Armenia | n.n. | - |  | Karagyan et al. 2011 |
| *Sphenoptera hispidula* Reitter, 1890 | Armenia | n.n. | - |  | Karagyan et al. 2011 |
| *Sphenoptera incerta (dolens)* Jakovlev, 1887 | Armenia | n.n. | - |  | Karagyan et al. 2011 |
| *Sphenoptera mesopotamica* Marseul, 1866 | Armenia | n.n. | - |  | Karagyan et al. 2011 |
| *Sphenoptera scovitzii* (Faldermann, 1835) | Armenia | n.n. | - |  | Karagyan et al. 2011 |
| *Sphenoptera smyrneensis* Gory, 1841 | Armenia | n.n. | - |  | Karagyan et al. 2011 |
| *Sphenoptera tamarisci beckeri* Dohrn, 1866 | Armenia | n.n. | - |  | Karagyan et al. 2011 |
| *Sphenoptera tragacanthae (glabrata)* (Klug, 1829) | Armenia | n.n. | - |  | Karagyan et al. 2011 |
| *Trachypteris (Melanophila)picta* (Pallas, 1773) | Morocco | n.n. | - |  | Karagyan et al. 2011 |
| *Trachypteris (Melanophila)picta decostigma* (Fabricius, 1787) | Armenia | n.n. | - |  | Karagyan et al. 2011 |
| *Trachys fragariae* Brisout, 1874 | Germany (Wurmlingen) | 01/06/2002 | - |  | Bernhard et al. 2005 |
| *Trachys minutus* (Linné, 1758) | Germany (Bienwald) | 25/04/2002 | A | KT199180 | Bernhard et al. 2005 |
| *Trachys phlyctaenoides* Kolenati, 1846 | Armenia | n.n. | - |  | Karagyan et al. 2011 |
| *Trachys scrobiculatus* Kiesenwetter, 1857 | Germany (Tübingen) | 18/06/2002 | - |  | Bernhard et al. 2005 |
| *Trachys troglodytes* Gyllenhal, 1817 | Germany (Hirschhalde) | 11/06/2002 | - |  | Korte et al. 2004 |
| **Hydraenidae** |  |  |  |  |  |
| *Hydraena antiatlantica* Jäch, Aguilera & Hernando, 1998 | n.n. | 01/11/2001 | - |  | this study |
| *Hydraena brachymera* d'Orchymont 1936 | n.n. | 31/10/2001 | A | KT199183 | this study |
| *Hydraena corinna* d’Orchymont, 1936 | n.n. | 30/10/2001 | A | KT199166 | this study |
| *Hydraena exasperata* d’Orchymont, 1935 | n.n. | 31/10/2001 | - |  | this study |
| *Hydraena gracilis* Germar, 1824 | n.n. | n.n. | A | KT199165 | Korte et al. 2004 |
| *Hydraena iberica* d’Orchymont, 1936 | n.n. | 31/10/2001 | A | KT199172 | Korte et al. 2004 |
| *Hydraena inapicipalpis* Pic 1918 | n.n. | 30/10/2001 | A | KT199164 | this study |
| *Hydraena lapidicola* Kiesenwetter, 1849 | n.n. | 09/11/2001 | - |  | this study |
| *Hydraena morio* Kiesenwetter, 1849 | n.n. | 09/11/2001 | A | KT199167 | this study |
| *Hydraena nigrita* Germar, 1824 | n.n. | 04/09/2001 | - |  | this study |
| *Hydraena pygmaea* Waterhouse, 1833 | n.n. | 31/07/2001 | A | KT199168 | this study |
| *Hydraena riberai* Jäch, Aguilera & Hernando, 1998 | n.n. | 01/11/2001 | A | KT199171 | this study |
| *Hydraena testacea* Curtis, 1830 | Spain | 31/10/2001 | - |  | this study |
| *Hydraena testacea* Curtis, 1830 | Morocco | 05/11/2001 | - |  | this study |
| *Hydraena testacea* Curtis, 1830 | n.n. | n.n. | - |  | this study |
| *Hydraena truncata* Rey, 1885 | n.n. | 09/11/2001 | A | KT199170 | this study |
| *Hydraenopsis sp.* | n.n. | n.n. | B | KT199198 | this study |
| *Limnebius aluta* Bedel, 1881 | n.n. | 09/11/2001 | - |  | this study |
| *Limnebius atomus* (Duftschmid, 1805) | n.n. | 01/11/2001 | B | KT199192 | this study |
| *Limnebius lusitanus* Balfour-Browne, 1979 | n.n. | 31/10/2001 | - |  | this study |
| *Limnebius mesatlanticus* Théry, 1933 | n.n. | 05/11/2001 | - |  | this study |
| *Limnebius truncatellus* (Thunberg, 1794) | n.n. | n.n. | A | KT199173 | this study |
| *Ochthebius exculptus* Germar, 1824 | n.n. | n.n. | B | KT199191 | this study |
| *Ochthebius lividipennis* Peyron, 1858 | n.n. | 09/11/2001 | B | KT199195 | this study |
| *Ochthebius melanescens* Dalla Torre, 1877 | n.n. | n.n. | - |  | Korte et al. 2004 |
| *Ochthebius meridionales* Rey, 1885 | n.n. | 09/11/2001 | B | KT199197 | this study |
| *Ochthebius minimus* (Fabricius, 1792) | n.n. | n.n. | B | KT199196 | Korte et al. 2004 |
| *Ochthebius quadrifoveolatus* Wollaston, 1854 | n.n. | 05/11/2001 | - |  | this study |
| *Ochthebius tivelunus* Ferro, 1984 | n.n. | 06/11/2001 | A | KT199174 | this study |
| **Dytiscidae** |  |  |  |  |  |
| *Acilius canaliculatus* (Nicolai, 1822) | Germany (Leipzig) | 22/06/2001 | - |  | this study |
| *Agabus bipustulatus* (Linnaeus, 1767) | Germany (Eilenburg) | 08/06/2001 | A | KT199169 | this study |
| *Agabus sturmi* (Gyllenhal, 1808) | Germany (Albrechtshain b. L.) | 30/04/2001 | - |  | this study |
| *Agabus uliginosus* (Linnaeus 1761) | Germany (Naunhof) | 26/05/2001 | - |  | this study |
| *Agabus undulatus* (Schrank, 1776) | Germany (Eilenburg) | 08/06/2001 | - |  | this study |
| *Agabus undulatus* (Schrank, 1776) | Germany (Ilmenau) | 04/07/2001 | - |  | this study |
| *Colymbetes fuscus* (Linnaeus, 1758) | Germany (Eilenburg) | 08/06/2001 | - |  | this study |
| *Copelatus haemorrhoidalis* (Fabricius, 1787) | Germany (Bavaria) | n.n. | A | KT199184 | this study |
| *Cybister lateralimarginalis* (De Geer, 1774) | Ungarn (Kiskunsági) | 26/05/2001 | - |  | this study |
| *Hydroporus dorsalis* (Fabricius, 1787) | Germany (Eilenburg) | 08/06/2001 | - |  | this study |
| *Hydroporus palustris* (Linnaeus, 1761) | Germany (Ilmenau) | 05/07/2001 | - |  | this study |
| *Hydroporus palustris* (Linnaeus, 1761) | Germany (Niederspree) | 11/07/2001 | - |  | this study |
| *Hydroporus planus* (Fabricius, 1781) | Germany (Naunhof) | 26/06/2001 | - |  | this study |
| *Hygrotus inaequalis* (Fabricius, 1777) | Germany (Ilmenau) | 04/07/2001 | - |  | this study |
| *Hygrotus versicolor* (Schaller, 1783) | Germany (Leipzig) | 30/04/2001 | B | KT199190 | this study |
| *Hyphydrus ovatus* (Linnaeus, 1761) | Germany (Leipzig) | 03/05/2001 | - |  | this study |
| *Hyphydrus ovatus* (Linnaeus, 1761) | Germany (Leipzig) | 01/05/2001 | - |  | this study |
| *Ilybius fuliginosus* (Fabricius, 1792) | Germany (Leipzig) | 22/06/2001 | - |  | this study |
| *Ilybius obscurus* (Marsham, 1802) | Germany (Niederspree) | 11/07/2001 | - |  | this study |
| *Laccophilus minutus* (Linnaeus, 1758) | Germany (Naunhof) | 26/05/2001 | - |  | this study |
| *Laccophilus hyalinus* (De Geer, 1774) | Germany (Leipzig) | 30/04/2001 | - |  | this study |
| *Platambus maculatus* (Linnaeus, 1758) | Germany (Leipzig) | 20/04/2001 | - |  | this study |
| *Platambus maculatus* (Linnaeus, 1758) | Germany (Ilmenau) | 05/07/2001 | - |  | this study |
| *Rhantus notatus* (Fabricius, 1781) | Germany (Eilenburg) | 08/06/2001 | - |  | this study |
| *Rhantus pulverosus* (Stephens, 1828) | Germany (Leipzig) | 22/06/2001 | - |  | this study |
| **Hydrophilidae** |  |  |  |  |  |
| *Berosus luridus* (Linneaus, 1776) | n.n. | n.n. | A | KT199182 | Bernhard et al. 2005 |
| *Coelostoma orbiculare* (Fabricius, 1775) | n.n. | n.n. | - |  | Korte et al. 2004 |
| *Enochrus quadripunctatus* (Herbst, 1797) | n.n. | n.n. | - |  | Bernhard et al. 2006 |
| *Enochrus sp.* | n.n. | n.n. | - |  | Bernhard et al. 2009 |
| *Enochrus sp.* | n.n. | n.n. | - |  | Bernhard et al. 2010 |
| *Enochrus sp.* | n.n. | n.n. | - |  | Bernhard et al. 2011 |
| *Helophorus aquaticus* (Linnaeus, 1758) | n.n. | n.n. | - |  | Bernhard et al. 2006 |
| *Helophorus aquaticus* (Linnaeus, 1758) | n.n. | n.n. | - |  | Bernhard et al. 2006 |
| *Helophorus nivalis* Giraud, 1851 | n.n. | n.n. | - |  | Bernhard et al. 2006 |
| *Hydrobius fuscipes* (Linnaeus, 1758) | n.n. | n.n. | - |  | Bernhard et al. 2006 |
| *Hydrochara caraboides* (Linné, 1758) | n.n. | n.n. | - |  | this study |
| *Hydrochus carinatus* Germar, 1824 | n.n. | n.n. | - |  | Bernhard et al. 2009 |
| *Hydrochus sp.* | n.n. | n.n. | A | KT199177 | Bernhard et al. 2009 |
| *Laccobius minutus* (Linné,1758) | n.n. | n.n. | - |  | this study |
| *Sphaeridium bipustulatum* (Fabricius, 1781) | n.n. | n.n. | - |  | Korte et al. 2004 |
| **Gyrinidae** |  |  |  |  |  |
| *Gyrinus sp..* | Germany (Leipzig) | 15/05/2001 | - |  | this study |
| *Gyrinus substriatus* Stephens, 1828 | Germany (Wildenhainer Bruch) | 15/04/2001 | - |  | this study |
| *Gyrinus suffriani* Scriba, 1855 | Germany (Leipzig) | 30/04/2001 | A | KT199176 | this study |
| **Haliplidae** |  |  |  |  |  |
| *Haliplus flavicollis* Sturm, 1834 | Germany (Leipzig) | 03/05/2001 | - |  | this study |
| *Haliplus fluviatilis* Aubé, 1836 | Germany (Niederspree) | 11/07/2001 | A | KT199178 | this study |
| *Haliplus flavicollis* Sturm, 1834 | Germany (Niederspree) | 11/07/2001 | - |  | this study |
| **Noteridae** |  |  |  |  |  |
| *Noterus clavicornis* (De Geer, 1774) | Germany (Leipzig) | 03/05/2001 | A | KT199175 | this study |
| *Noterus sp.* | Germany (Bad Dürenberg) | 01/05/2001 | A | KT199179 | this study |
|  |  |  |  |  |  |
| **References** |  |  |  |  |  |
| Bernhard D, Fritzsch G, Glockner P, Wurst C (2005) Molecular insights into speciation in the Agrilus viridis-complex and the genus Trachys (Coleoptera: Buprestidae). European Journal of Entomology 102(4), 599–605. | | | | | |
| Bernhard D, Schmidt C, Korte A, Fritzsch G, Beutel RG (2006) From terrestrial to aquatic habitats and back again – molecular insights into the evolution and phylogeny of Hydrophiloidea (Coleoptera) using multigene analyses. Zoologica Scripta 35(6), 597–606. | | | | | |
| Bernhard D, Komarek A, Beutel R, Ribera I (2009) Phylogenetic analysis of Hydrophiloidea (Coleoptera: Polyphaga) based on molecular data and morphological characters of adults and immature stages. Insect Systematics & Evolution 40(1), 3–41. | | | | | |
| Korte A, Ribera I, Beutel R, Bernhard D (2004) Interrelationships of Staphyliniform groups inferred from 18S and 28S rDNA sequences, with special emphasis on Hydrophiloidea (Coleoptera, Staphyliniformia). Journal of Zoological Systematics and Evolutionary Research 42(4), 281–288. | | | | | |
| Karagyan, G., Bernhard, D., Hering, L., Perseke, M., Kalashian, M. (2011) Phylogenetic relationships within the Buprestidae (Insecta: Coleoptera) based on molecular studies. In: Proceedings of the international conference "Biological diversity and conservation problems of the fauna of the Caucasus", Yerevan, Armenia. | | | | | |
