# Supplementary material for: Wolbachia distribution in selected beetle taxa characterized by PCR screens and MLST data
Source: Ecol Evol. 2015 Sep 16;5(19):4345–53. doi: 10.1002/ece3.1641 (PMC4667820; doi:10.1002/ece3.1641)
Supplement: Supplementary file 3 — Table S2. NCBI Accession numbers for all sequences employed for Hydraenidae and Wolbachia MLST phylogeny. [file ECE3-5-4345-s003.doc]

| Table S2 NCBI Accession numbers for all sequences employed for Hydraenidae and Wolbachia MLST phylogeny. Sequences generated in this study are highlighted in bold. Slashes indicate failed PCRs, dashes indicate Wolbachia- negative species. | | | | | | | | |
| --- | --- | --- | --- | --- | --- | --- | --- | --- |
|  | | | | | | | | |
| Species | *COI* | *18S-rDNA* | *28S-rDNA* | *coxA* | *gatB* | *fbpA* | *ftsZ* | *hcpA* |
| *Hydraena antiatlantica* | HM588387 | HM588593 | HM588542 | - | - | - | - | - |
| *Hydraena brachymera* | HE970832 | **KT199105** | **KT199117** | **KT199137** | **KT199204** | / | **KT199183** | **KT199218** |
| *Hydraena corinna* | HM588334 | HM588567 | HM588516 | **KT199138** | **KT199205** | **KT199151** | **KT199166** | **KT199219** |
| *Hydraena exasperata* | HM588382 | HM588590 | HM588539 | - | - | - | - | - |
| *Hydraena gracilis* | HM588384 | HM588575 | HM588524 | / | / | / | **KT199165** | / |
| *Hydraena iberica* | HM588403 | AJ810730 | AJ810765 | **KT199139** | **KT199206** | **KT199152** | **KT199172** | / |
| *Hydraena inapicipalpis* | HE970811 | HE970943 | HE970989 | **KT199140** | **KT199207** | **KT199153** | **KT199164** | **KT199220** |
| *Hydraena lapidicola* | HM588337 | **KT199106** | **KT199118** | - | - | - | - | - |
| *Hydraena morio* | HM588320 | HM588558 | HM588507 | **KT199141** | **KT199208** | **KT199154** | **KT199167** | **KT199221** |
| *Hydraena nigrita* | HM588352 | HM588576 | HM588525 | - | - | - | - | - |
| *Hydraena pygmaea* | HM588353 | HM588577 | HM588526 | **KT199142** | **KT199209** | **KT199155** | **KT199168** | / |
| *Hydraena riberai* | HM588388 | HM588594 | HM588543 | **KT199143** | **KT199210** | **KT199156** | **KT199171** | **KT199222** |
| *Hydraena testacea* | HM588386 | HM588592 | HM588541 | - | - | - | - | - |
| *Hydraena truncata* | HM588383 | HM588579 | HM588528 | **KT199144** | **KT199211** | **KT199157** | **KT199170** | **KT199223** |
| *Hydraenopsis sp.* | HM588391 | HM588582 | HM588531 | **KT199145** | **KT199212** | **KT199158** | **KT199198** | **KT199224** |
| *Limnebius lusitanus* | **KT199131** | **KT199109** | **KT199121** | - | - | - | - | - |
| *Limnebius mesatlanticus* | **KT199132** | **KT199110** | **KT199122** | - | - | - | - | - |
| *Limnebius aluta* | **KT199129** | **KT199107** | **KT199119** | - | - | - | - | - |
| *Limnebius atomus* | **KT199130** | **KT199108** | **KT199120** | / | / | / | **KT199192** | / |
| *Limnebius truncatellus* | HQ16529 | **KT199111** | **KT199123** | **KT199146** | **KT199213** | **KT199159** | **KT199173** | **KT199225** |
| *Ochthebius exsculptus* | GU143760 | **KT199112** | **KT199124** | / | / | / | **KT199191** | / |
| *Ochthebius lividipennis* | KT199133 | **KT199113** | **KT199125** | **KT199147** | **KT199214** | **KT199160** | **KT199195** | **KT199226** |
| *Ochthebius melanescens* | GU143748 | AJ810732 | AJ810767 | - | - | - | - | - |
| *Ochthebius meridionales* | **KT199134** | **KT199114** | **KT199126** | **KT199148** | **KT199215** | **KT199161** | **KT199197** | **KT199227** |
| *Ochthebius minimus* | DQ155775 | HE970955 | HE970995 | **KT199149** | **KT199216** | **KT199162** | **KT199196** | **KT199228** |
| *Ochthebius qaudrifo veolatus* | **KT199135** | **KT199115** | **KT199127** | - | - | - | - | - |
| *Ochthebius tivelunus* | **KT199136** | **KT199116** | **KT199128** | **KT199150** | **KT199217** | **KT199163** | **KT199174** | **KT199229** |
| *Ptenidium sp.* | HE970847 | HE970959 | HE970997 | - | - | - | - | - |
| *Ptiliolum sp.* | HE970857 | HE970967 | HE971004 | - | - | - | - | - |
