# Supplementary material for: Wolbachia distribution in selected beetle taxa characterized by PCR screens and MLST data
Source: Ecol Evol. 2015 Sep 16;5(19):4345–53. doi: 10.1002/ece3.1641 (PMC4667820; doi:10.1002/ece3.1641)
Supplement: Supplementary file 4 — Table S3. Results of BaTS analyses for phylogeny‐trait associations. [file ECE3-5-4345-s004.doc]

**Table S3** Results of BaTS analyses for phylogeny-trait associations. For each of the tested phylogenies, observed and expected values, and the level of significance are given for three different test statistics. P-values < 0.05 are highlighted in bold.

|  | Observed mean | Lower 95% CI | Upper 95% CI | Null mean | Lower 95% CI | Upper 95% CI | **P-value** |  |
| --- | --- | --- | --- | --- | --- | --- | --- | --- |
| **ClonalFrame** |  |  |  |  |  |  |  |  |
| AI | 0.07 | 0.06 | 0.08 | 0.92 | 0.57 | 1.22 | **0.001** |  |
| PS | 3.00 | 3.00 | 3.00 | 5.56 | 4.55 | 6.00 | **0.002** |  |
| MC Hydraena | 4.64 | 4.00 | 5.00 | 2.43 | 1.21 | 4.62 | **0.001** |  |
| MC Ochthebius | 3.00 | 3.00 | 3.00 | 1.24 | 1.00 | 2.00 | **0.026** |  |
| **MrBayes all taxa** |  |  |  |  |  |  |  |  |
| AI | 1.73 | 1.54 | 1.88 | 2.12 | 1.43 | 2.71 | 0.206 |  |
| PS | 10.00 | 10.00 | 10.00 | 12.41 | 10.04 | 14.32 | **0.043** |  |
| MC supergroup A | 2.03 | 2.00 | 2.00 | 2.02 | 1.00 | 2.79 | 0.718 |  |
| MC supergroup B | 2.00 | 2.00 | 2.00 | 1.26 | 1.00 | 2.00 | 0.171 |  |
| MC no Wolbachia | 2.00 | 2.00 | 2.00 | 2.27 | 1.58 | 4.00 | 0.863 |  |
| **MrBayes Wolbachia positive taxa** |  |  |  |  |  |  |  |  |
| AI | 0.27 | 0.13 | 0.30 | 0.85 | 0.40 | 1.29 | **0.023** |  |
| PS | 2.00 | 2.00 | 2.00 | 4.20 | 3.00 | 5.00 | **0.006** |  |
| MC supergroup A | 9.00 | 9.00 | 9.00 | 3.45 | 2.00 | 7.00 | **0.009** |  |
| MC supergroup B | 2.00 | 2.00 | 2.00 | 1.44 | 1.00 | 2.02 | 0.268 |  |

AI - Association Index (Wang et al. 2001)

CI - Confidence interval

PS - Parsimony score (Slatkin & Maddison)

MC - Maximum exclusive single-state clade size (Parker et al. 2008)

References

Parker, J., Rambaut, A.R. & Pybus, O.G. (2008) Correlating viral phenotypes with phylogeny: accounting for

phylogenetic uncertainty. MEEGID 8(3):239-246.

Slatkin, M., & Maddison, W.P. (1989). A cladistic measure of gene flow measured from the phylogenies of

alleles. Genetics 123(3):603-613.

Wang, T.H., Donaldson, Y.K., Brettle, R.P., Bell, J.E. & Simmonds, P. (2001). Identification of shared populations

of Human immunodeficiency Virus Type 1 infecting microglia and tissue macrophages outside the central

nervous system. J. Virol. 75 (23): 11686-11699.
