# Supplementary material for: Wolbachia distribution in selected beetle taxa characterized by PCR screens and MLST data
Source: Ecol Evol. 2015 Sep 16;5(19):4345–53. doi: 10.1002/ece3.1641 (PMC4667820; doi:10.1002/ece3.1641)
Supplement: Supplementary file 5 — Table S4. Results of ParaFit and PACo tests for congruence of Hydraenidae and Wolbachia phylogenies. [file ECE3-5-4345-s005.doc]

**Table S4** Results of ParaFit and PACo tests for congruence of Hydraenidae and *Wolbachia* phylogenies

| Distance matrix used | ParaFit | | PACo | |
| --- | --- | --- | --- | --- |
|  | ParaFitGlobal | p-value | m2 | p-value |
| Genetic distances | 0.001 | 0.031 | 0.030 | 0.004 |
| Patristic distances (branch lengths) | 0.006 | 0.003 | 0.101 | 0.002 |
